# The effect of nanometre-scale V-pits on electronic and optical properties and efficiency droop of GaN-based green light-emitting diodes

**DOI:** 10.1038/s41598-018-29440-4

**Published:** 2018-07-23

**Authors:** Shengjun Zhou, Xingtong Liu, Han Yan, Yilin Gao, Haohao Xu, Jie Zhao, Zhijue Quan, Chengqun Gui, Sheng Liu

**Affiliations:** 1 Hubei Key Laboratory of Accoutrement Technique in Fluid Machinery and Power Engineering, School of Power and Mechanical Engineering, Wuhan University, Wuhan, 430072 China; 2 Research Center of Electronic Manufacturing and Packaging Integration, Institute of Technological Sciences, Wuhan University, Wuhan, 430072 China; 3 National Institute of LED on Si Substrate, Nanchang University, Nanchang, 330047 China; 4 School of Mechanical and Electrical Engineering, Wuhan University of Technology, Wuhan, 430070 China

## Abstract

The development of efficient green light-emitting diodes (LEDs) is of paramount importance for the realization of colour-mixing white LEDs with a high luminous efficiency. While the insertion of an InGaN/GaN superlattice (SL) with a lower In content before the growth of InGaN/GaN multiple quantum wells (MQWs) is known to increase the efficiency of LEDs, the actual mechanism is still debated. We therefore conduct a systematic study and investigate the different mechanisms for this system. Through cathodoluminescence and Raman measurements, we clearly demonstrate that the potential barrier formed by the V-pit during the low-temperature growth of an InGaN/GaN SL dramatically increases the internal quantum efficiency (IQE) of InGaN quantum wells (QWs) by suppressing non-radiative recombination at threading dislocations (TDs). We find that the V-pit potential barrier height depends on the V-pit diameter, which plays an important role in determining the quantum efficiency, forward voltage and efficiency droop of green LEDs. Furthermore, our study reveals that the low-temperature GaN can act as an alternative to an InGaN/GaN SL structure for promoting the formation of V-pits. Our findings suggest the potential of implementing optimized V-pits embedded in an InGaN/GaN SL or low-temperature GaN structure as a beneficial underlying layer for the realization of highly efficient green LEDs.

## Introduction

In recent years, white light sources based on the colour mixing of red, green, and blue light-emitting diodes (LEDs) have attracted much interest due to their advantages of a high colour-rendering index and high luminous efficiency^1–7^. The external quantum efficiency (EQE) of blue InGaN/GaN LEDs can exceed 80%. However, high-In-content green InGaN/GaN LEDs have a relatively low efficiency, which is well known as the ‘green gap’ phenomenon^8–14^. The efficiency of a colour-mixing white light source is therefore limited by the efficiency of the green LEDs. As a result, it is important to improve the efficiency of green LEDs for the realization of highly efficient colour-mixing white LEDs.

Due to the large lattice constant and thermal expansion coefficient mismatches between GaN and the sapphire substrate, the threading dislocation (TD) density in GaN-based LEDs is on the order of 10^8^ cm^−2^^15–20^. These dislocations typically act as non-radiative recombination centres (NRCs) and carrier leakage paths, thereby giving rise to a low emission efficiency^21^. A second issue related to obtaining high-efficiency green LEDs is the strong polarization effects that arise from their wurtzite crystal structure. In particular, piezoelectric and spontaneous polarization effects generate strong internal electric fields in the InGaN/GaN multiple quantum well (MQW), leading to a spatial separation of the electron and hole wave functions in the quantum wells (QWs) and, thus, a reduction in the radiative recombination rates^22,23^.

The insertion of an InGaN or InGaN/GaN superlattice (SL) underlayer in the epi-structure is known to strongly improve the efficiency of GaN-based LEDs. The inclusion of such a layer has been extensively reported in the literature, and several mechanisms have been proposed to explain the improvement in LED efficiency^24–33^. Leem *et al*. suggested that the InGaN/GaN SL acts as a strain relief layer, lowering the internal piezoelectric field and thus alleviating the detrimental impact of the quantum confined Stark effect (QCSE)^25^. Otsuji *et al*. and Ju *et al*. reported that an InGaN or InGaN/GaN underlayer can behave as an electron reservoir, leading to an enhancement of the electron capture efficiency^26,27^. Akasaka *et al*. and Törmä *et al*. reported that the InGaN underlayer strongly reduces the density of the defect-related NRCs that affect the MQW active region^28–30^. However, Takahashi *et al*. reported that the inclusion of low-temperature-grown GaN beneath InGaN/GaN QWs can also give rise to an improved LED emission efficiency^31^. This finding is somewhat contradictory to the results of Akasaka *et al*. and Törmä *et al*. Davies *et al*. proposed that the inclusion of a Si–doped InGaN underlayer and the subsequent pinning of the Fermi level near the conduction band edge can increase the surface polarization field, which in turn reduces the built-in electric field in the QWs and thus leads to an increase in the QW radiative efficiency^32^. However, the results of an investigation of Haller *et al*. revealed that the InGaN underlayer should no longer influence the oscillator strength for GaN spacers thicker than 20 nm (corresponding to the distance between the MQW and the InGaN underlayer)^33^.

Another proposed mechanism involves the formation of V-pits at the dislocation surface termination during the low-temperature growth of an InGaN/GaN SL or GaN layer. Hangleiter *et al*. and Tomiya *et al*. demonstrated that {10–11} QWs exist at the sidewall of the V-pits, and that the thickness and In concentration of the {10–11} QWs were smaller and lower than those of *c*-plane QWs^34,35^. These results revealed that the V-pits around TDs might act as energy barriers that play a critical role in the suppression of the lateral transport of the carriers into TDs and thus prevent the carriers from recombining nonradiatively at the TDs^36–38^. Recently, by performing spatially resolved photoluminescence (PL) measurements with scanning near-field optical microscopy, Okada *et al*. confirmed that the improvement in LED efficiency is due to the presence of the potential barrier formed by the V-pits around the TDs^39^. Multiple studies have explored the possibility of obtaining the optimal V-pit size for improving the efficiency of GaN-based LEDs by manipulating the periods of InGaN/GaN SL^40–42^. However, practical questions remain unanswered that are critical to device design and optimization, such as how V-pits affect the electronic and optical properties and efficiency droop of green LEDs.

In this study, we unambiguously demonstrate that the potential barrier formed by the V-pit dramatically increases the internal quantum efficiency (IQE) of the InGaN QWs by suppressing non-radiative recombination of the carriers at the TDs. We have analysed the effects of nanometre-scale V-pits on the electronic and optical properties and efficiency droop of green LEDs using various periods of InGaN/GaN SLs and low-temperature GaN. We found that the V-pit potential barrier height around the TD is determined by the size of the V-pit that plays an important role in determining the quantum efficiency, forward voltage and efficiency droop of green LEDs. These findings, obtained from green LEDs with various underlying layer configurations, including InGaN/GaN SL and low-temperature GaN, reveal several unexpected effects and are important for future device optimization.

## Methods

### Growth and device fabrication

The green MQW active region consisting of 9 pairs of In_0.25_Ga_0.75_N/GaN was grown by metal-organic chemical vapour deposition (MOCVD) on 0, 2, 4, 6, 8, and 10 periods of In_0.06_Ga_0.94_N/GaN SL with a thickness of approximately 4.2 nm and 31 nm or on a low-temperature GaN layer with a thickness equal to 6 periods of In_0.06_Ga_0.94_N/GaN SL. InGaN QWs with a nominal thickness of 3.5 nm were grown at 760 °C, after which a thin (approximately 1 nm thick) GaN capping layer was grown at the same temperature followed by a temperature ramp and growth of the remaining 13 nm GaN barrier layer at 920 °C. Trimethylaluminum (TMAl), trimethylgallium (TMGa), trimethylindium (TMIn), and ammonia (NH_3_) were used as the precursors for Al, Ga, In, and N, respectively. Silane (SiH_4_) and bis (cyclopentadienyl)magnesium (CP_2_ Mg) were used as the n-dopant and p-dopant source, respectively. Hydrogen (H_2_) was used as the carrier gas for the growth of the GaN epilayer, while nitrogen (N_2_) was used as the main carrier gas for the InGaN epilayer growth.

The full green LEDs, which were initiated with the deposition of a 25 nm thick Al_0.1_Ga_0.9_N nucleation layer on a sapphire substrate in a VEECO K465i (Veeco, NY, USA) reactor, were composed of a 2.5 *μ*m thick undoped GaN layer grown at 1065 °C, a 90 nm thick n-Al_0.15_Ga_0.85_N layer (Si doping = 2 × 10^18^ cm^−3^) at 980 °C, a 2.5 *μ*m thick heavily Si–doped n+-GaN layer (Si doping = 2 × 10^19^ cm^−3^) at 1075 °C, a 200 nm thick lightly Si–doped n–GaN layer (Si doping = 5 × 10^17^ cm^−3^) at 1075 °C, various periods (0, 2, 4, 6, 8, and 10) of In_0.06_Ga_0.94_N (4 nm)/GaN (31 nm) SL at 800 °C or 210 nm thick low-temperature GaN at 800 °C, 9 pairs of In_0.25_Ga_0.75_N/GaN MQW, a 75 nm thick p-GaN layer at 780 °C, a 72 nm thick structure of 18 periods of p-Al_0.2_Ga_0.8_N/GaN SL (Mg doping = 1.7 × 10^20^ cm^−3^) at 850 °C, a 340 nm thick p-GaN layer (Mg doping = 6 × 10^19^ cm^−3^) at 950 °C, and a 10 nm thick heavily Mg–doped p+-GaN layer (Mg doping = 1.6 × 10^20^ cm^−3^) at 700 °C. For the seven green LED samples investigated, all growth parameters of the green LED structure were kept constant except for the underlying SL and low-temperature GaN used for comparison.

**Figure 1:**
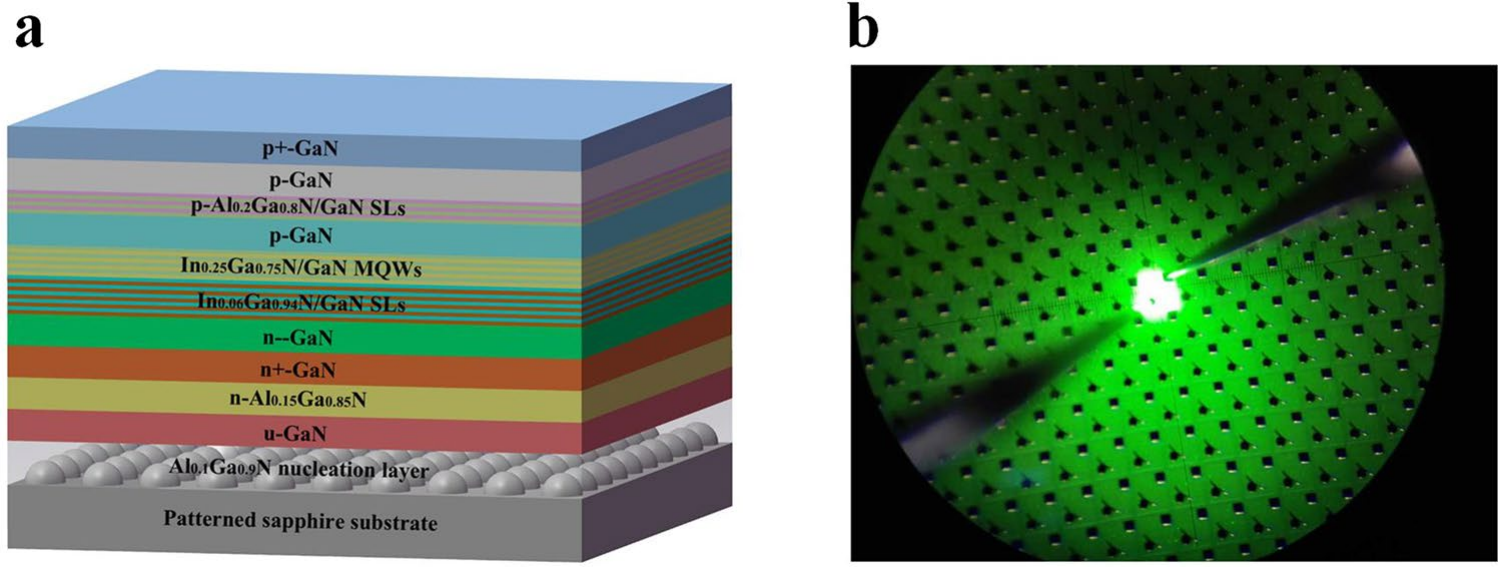
(**a**) Schematic illustration of the green LED structure. (**b**) Electroluminescence image of a green LED at 20 mA.

For the fabrication of the green LED chips, a mesa depth of 1.5 *μ*m was defined by an inductively coupled plasma process based on the BCl_3_/Cl_2_ mixture gas. A 230 nm thick ITO layer was deposited as the current spreading layer on the p-GaN layer, followed by thermal annealing in N_2_ ambient at 540 °C for 20 min to improve the ohmic contact between the ITO and p-GaN. Cr/Pt/Au metallization layers were then evaporated as n-type and p-type electrodes. Finally, the green LED wafers were diced into chips with dimensions of 305 × 330 *μ*m^2^. The light intensity versus current characteristics and current versus voltage characteristics of the green LEDs were measured using a probe station system. Figure 1 shows a schematic illustration of the full green LED structure and the electroluminescence (EL) image of a green LED chip measured at 20 mA.

### Measurement and characterization

Atomic force microscopy (AFM) images were obtained using a Bruker Multimode 8 in tapping mode. Cathodoluminescence (CL) experiments were performed using an FEI Quanta 200 F field emission scanning electron microscope (SEM) fitted with a Gatan Mono CL3+ system under a vacuum of 10^−6^ Torr and at an acceleration voltage of 2.0 kV and room temperature. Transmission electron microscopy (TEM) images were taken with an FEI Tecnai F20 system at 200 kV, and both cross-sectional and plan-view TEM samples were prepared by focus ion beam milling using Ga ions at 30 kV. The Ga, N, In, and Al contents were measured using energy dispersive X-ray (EDX) spectroscopy.

### First-principles calculations

The surface energies of GaN(10–11) and GaN(0001) were calculated using first-principles molecular dynamics calculations. The surfaces were modelled by repeated cells that typically contain 12 Ga and 12 N atoms in each unit cell, and then a single Ga atom was replaced by one In atom to denote the InGaN alloys with a specific In composition. In the present calculations, the interactions of the ion cores with the valence electrons were described by ultrasoft pseudopotentials^43^. The PBE exchange functional^44^ was used as the implementation of the generalized gradient approximation (GGA) approach. Full atomic relaxations were performed for all structures. The calculations were performed in the NPT ensemble with an energy tolerance of 2 × 10^−6^ eV/atom. Periodic boundary conditions were applied along the crystallographic axial directions.

## Results and Discussion

**Figure 2:**
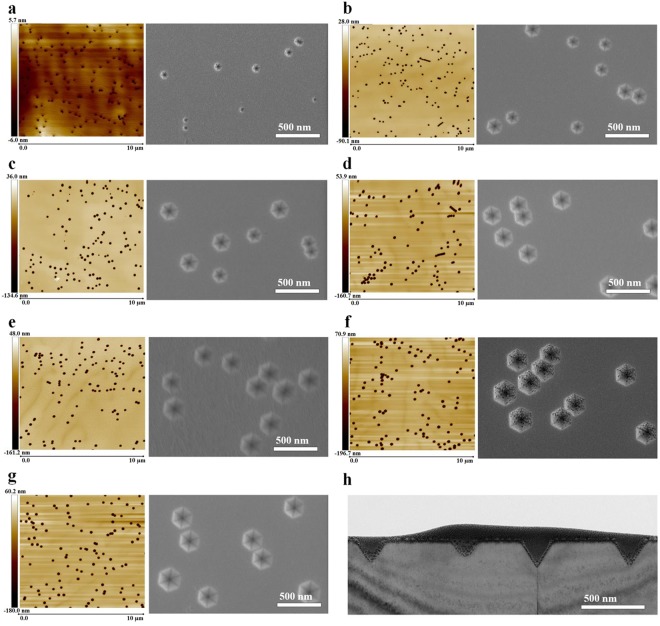
10 × 10 μm^2^ AFM scans and top-view SEM images of the green In_0.25_Ga_0.75_N/GaN MQWs grown on (**a**) 0, (**b**) 2, (**c**) 4, (**d**) 6, (**e**) 8, (**f**) 10 periods of In_0.06_Ga_0.94_N/GaN SL and on (**g**) a low-temperature GaN layer. The V-pit diameter is proportional to the number of SL periods. The thickness of the low-temperature GaN layer is equal to that of 6 periods of SL. (**h**) Cross-sectional TEM image of an MQW sample showing V-shaped pits.

Figure 2(a–g) show the AFM and morphological SEM images of the green In_0.25_Ga_0.75_N/GaN MQWs grown on the 0, 2, 4, 6, 8, and 10 periods of In_0.06_Ga_0.94_N/GaN SL and on low-temperature GaN. From the AFM scans of Fig. 2(a–g), a high density of dark pits can be observed, and the density of dark pits was determined to be 1.62 × 10^8^, 1.59 × 10^8^, 1.28 × 10^8^, 1.20 × 10^8^, 1.23 × 10^8^, 1.24 × 10^8^, and 1.21 × 10^8^ cm^−2^. The dark pits in the AFM image are found to be hexagonal pit structures in the SEM images. Figure 2(h) shows the cross-sectional TEM image of a green MQW sample, where the hexagonal pits have a V-shaped cross section, which constitutes the so-called V-pit. Here, the V-pit diameter was defined as the distance between the two parallel sides of the hexagonal V-pit. We found that the V-pit diameter depends on the number of SL periods. As the number of SL periods increases, the V-pit expands. The V-pit diameter of the green MQWs grown on 0, 2, 4, 6, 8, and 10 periods of SL and on low-temperature GaN was measured to be 99 nm, 157 nm, 185 nm, 207 nm, 225 nm, 252 nm, and 230 nm, respectively, indicating that the V-pit diameter increases with increasing SL periods.

Although the low-temperature GaN is as thick as 6 SL periods, the V-pit diameter of the green MQW grown on the low-temperature GaN (230 nm) is larger than that of the green MQW grown on 6 SL periods (207 nm). Wu *et al*. reported that the orientation of the inclined sidewall plane of V-pit that terminates the QWs is determined by the relative growth rate of the material in the V-pit in comparison with the growth on the adjacent (0001) plane^45^. During the MOCVD growth process, we note that the growth rate of low-temperature GaN is higher than of SL. In the growth of the GaN layer at a low temperature, V-pits having {10–1*x*} facets with a higher index *x* are formed, thus enabling the formation of V-pits with larger size than those formed by {10–11} facets. We therefore conclude that the growth of GaN at low temperature is more effective for expanding the V-pit size than the growth of InGaN/GaN SL, revealing that low-temperature GaN could be an alternative to the InGaN/GaN SL for promoting the formation of V-pits.

**Figure 3:**
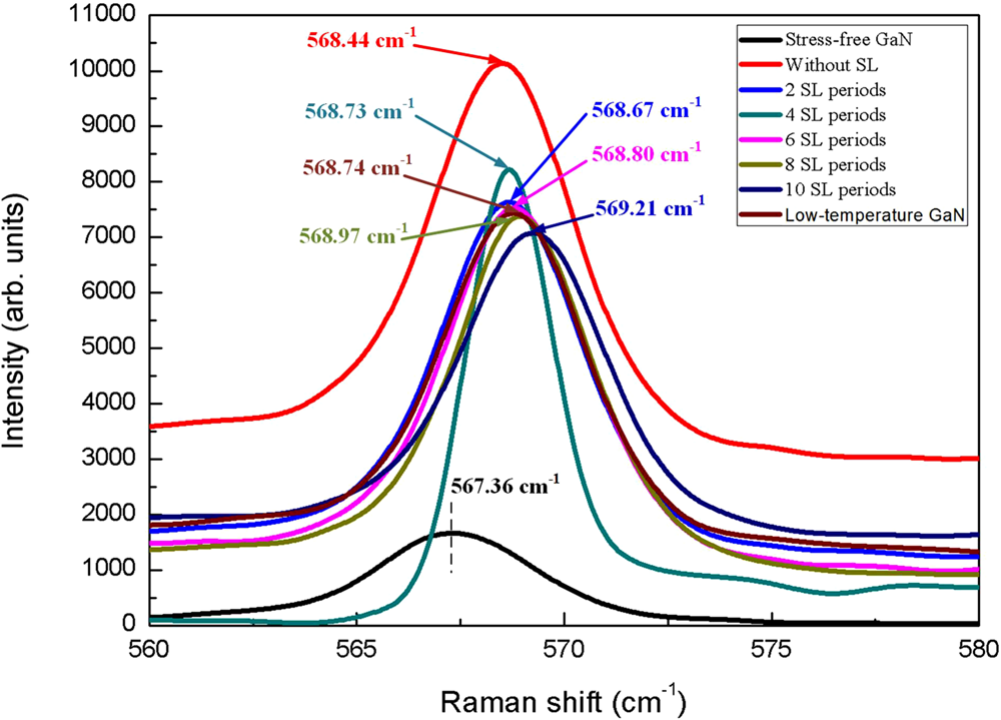
Raman spectra of green LEDs grown on 0, 2, 4, 6, 8, and 10 periods of SL and on low-temperature GaN. Dashed line indicates the stress-free GaN E_2_ peak at 567.36 cm^−1^.

The high-energy Raman E_2_ mode was measured using Raman spectroscopy to evaluate the stress in green LEDs. Figure 3 shows the Raman spectra of green LEDs grown on 0, 2, 4, 6, 8, and 10 periods of SL and on low-temperature GaN. In Fig. 3, the peaks for the green LEDs grown on 0, 2, 4, 6, 8, and 10 periods of SL and on low-temperature GaN were 568.44, 568.67, 568.73, 568.80, 568.97, 569.21, and 568.74 cm^−1^, respectively. Generally, the stress in the GaN layers can be calculated by $\sigma (Raman)=\frac{\Delta \omega }{4.2}Gpa$, where Δ*ω* is the frequency shift of the GaN high-energy E_2_ mode peak. In Fig. 3, the stress-free GaN E_2_ peak is located at 567.36 cm^−1^ (dashed line). The E_2_ peak of the green LEDs grown on the 0, 2, 4, 6, 8, and 10 periods of SL and on low-temperature GaN redshifts with respect to the stress-free GaN, indicating the presence of compressive strain in the green LEDs. According to the Δ*ω*, the compressive stress of green LEDs grown on 0, 2, 4, 6, 8, and 10 periods of SL and on low-temperature GaN was calculated to be 257, 312, 326, 343, 383, 440, and 329 MPa, respectively.

The compressive stress of the green LED with SL increased compared with that of the green LED without SL. Furthermore, as the number of SL periods increased, the compressive stress of the green LEDs also increased. Even though the thickness of the low-temperature GaN is equal to that of 6 SL periods, the compressive stress of green LEDs grown on low-temperature GaN was lower than that of green LEDs grown on 6 SL periods. One explanation for this effect is that the InGaN material with the larger lattice generates an additional compressive strain in green LEDs. Much of the prior work has proposed that an InGaN or InGaN/GaN SL underlayer act as a strain relief layer and thereby reduces the piezoelectric field^25,46–48^. However, our findings here do not support the idea of InGaN/GaN SL as a possible underlayer for stress relief since our Raman measurements revealed a larger compressive stress with increasing InGaN/GaN SL periods. This result might be associated with the significantly lower ratio of InGaN to GaN in our SL structure than in the previously reported InGaN/GaN SL structure.

**Figure 4:**
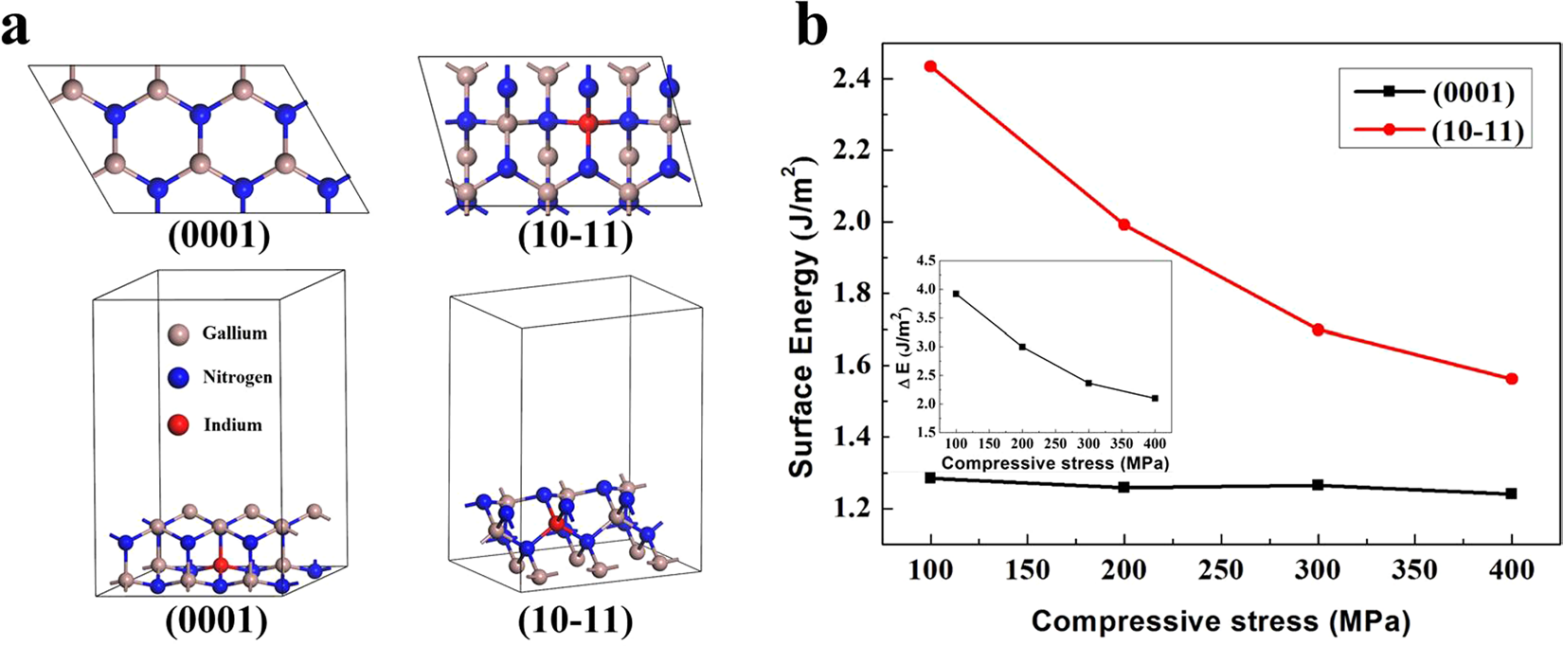
(**a**) Top and side views of the calculation model. (**b**) Surface energy of In-covered GaN(0001) and GaN(10–11) versus compressive stress.

To explain why the density of the V-pits decreases and the size of V-pits increases as the periods of SL increase, the surface energies of GaN (0001) and GaN (10–11) are calculated using first-principles molecular dynamics simulations. Figure 4(a) shows the top view and side view of the calculation model. Figure 4(b) shows the relationships between the surface energies and external stress. Due to the larger lattice of InGaN relative to that of GaN, the growth of thin InGaN on thick GaN is exposed to compressive stress. As shown in Fig. 3, the compressive stress in green LEDs increases with increasing SL periods. In the first-principles calculations, the surface energies of GaN (0001) and GaN (10–11) decrease with increasing compressive stress as shown in Fig. 4(b). This prediction is consistent with a previous report of the strain-dependent surface energies^49^. The decrease of the surface energy of GaN (10–11) caused by additional SL periods results in a higher surface diffusion rates and thus a lower V-pit density. Here we define the formation energies of the (0001) facet that is removed and (10–11) facets that are formed as Δ*E* = *E*_*surface*_ (10 − 11)/*α* − *E*_*surface*_ (0001), where *α* = 0.468 is the scalar product of the unit vectors that are normal to the (10–11) and (0001) surfaces^50^. Our first-principles calculations results indicate that the Δ*E* decreases with increasing compressive stress as shown in inset of Fig. 4(b). Northrup *et al*. reported that the equilibrium size of V-pit increased with a decrease in Δ*E*^51^. Accordingly, the increasing compressive stress generated from the increase of SL periods can also result in a larger size of V-pit. Our first-principles calculations results indicate that the formation energies decrease with increasing compressive stress, as shown in Fig. 4(b). Accordingly, the increasing compressive stress generated from additional SL periods can also result in a larger V-pit size.

**Figure 5:**
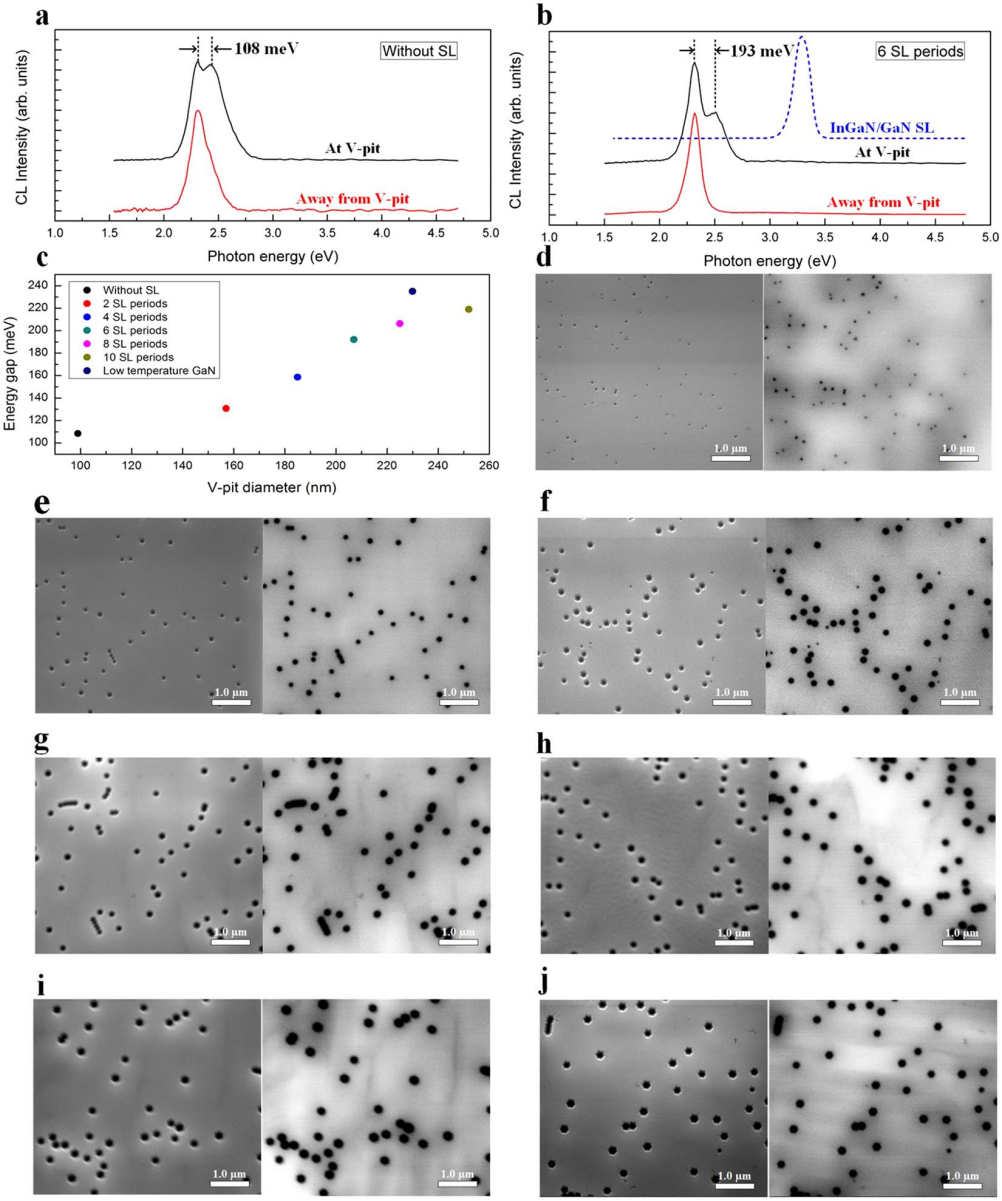
CL spectra measured from the green MQWs grown (**a**) without an SL and (**b**) on 6 SL periods and the sample with only SL grown on n-GaN layer. (**c**) V-pit diameter dependence of the energy gap between the main energy peak and the high-energy shoulder peak in CL spectra around the V-pits at 300 K. Top-view SEM and panchromatic CL images of the green MQWs grown (**d**) without an SL and on (**e**) 2 SL periods, (**f**) 4 SL periods, (**g**) 6 SL periods, (**h**) 8 SL periods, (**i**) 10 SL periods, and (**j**) a low-temperature GaN layer.

To gain further insights into the optical properties of the V-pit embedded green MQWs, the CL spectra of the green MQWs were measured around the V-pit and at a point away from the V-pit at an acceleration voltage of 2.0 kV at room temperature. Figure 5(a) shows the CL spectrum obtained around the V-pit on the green MQW sample without an SL, which exhibited two emission peaks, including the main energy peak and the high-energy shoulder peak. The main energy peak was approximately 2.31 eV, which was identical to that taken from the surrounding planar regions (away from the V-pit), and the high-energy shoulder peak was observed at approximately 2.42 eV. The energy gap between the main energy peak and the high-energy shoulder peak was approximately 108 meV. In addition, the CL spectrum around the V-pit on the green MQW sample grown on 6 periods of SL was also recorded [Fig. 5(b)], for which the energy gap between the main energy peak (2.32 eV) and the high-energy shoulder peak (2.51 eV) was approximately 193 meV. Taking into account the absorption of an emitted photon by the slowromancap 3@ -Nitride material with a typical absorption coefficient of about 10^5^ cm^−1^, the majority of the CL signal collected must arise from the first 25–30 nm of material below the surface^14,52^. However, there will be more electron penetration into the SL underlayer in the V-pit region than elsewhere due to the absence of material at the V-pit itself. Accordingly, although higher-energy emissions at the V-pits were observed, the possibility cannot be ruled out that the high-energy shoulder peak was related to the emission of the low-In-content InGaN/GaN SL underlayer. To exclude the possibility of high-energy shoulder peak arising from the SL underlayer, another sample with only In_0.06_Ga_0.94_N/GaN SL was grown on n-GaN layer for investigation of the CL emission peak of SL. The emission peak of SL centered at around 3.28 eV was completely unrelated to the high energy shoulder peak (2.42–2.58 eV), as shown in Fig. 5(b). Thus, the higher-energy shoulder peaks observed at the V-pits were corresponded to the emission energy of the sidewall MQW of the V-pit, and the energy gap between the main energy peak and the high-energy shoulder peak indicated that a potential barrier around the dislocation was formed by the V-pit.

A detailed study of the energy gap dependence on the V-pit diameter is shown in Fig. 5(c). For these green MQWs grown on 0, 2, 4, 6, 8, and 10 periods of SL and on low-temperature GaN, the energy gap between the main energy peak and the high-energy shoulder peak was found to be 108, 131, 159, 193, 206, 219, and 235 meV, respectively. As shown in Fig. 5(c), the V-pit potential barrier height was almost saturated as the number of SL periods was increased from 6 to 10. In addition, the V-pit potential barrier height of the green MQW grown on low-temperature GaN was higher than that of the green MQWs grown on various periods of SL, which might be attributed to the change in the migration, diffusion, and adsorption of adatoms around the V-pit and the border between the original (0001) *c*-plane and {10 − 1*x*} facets in the V-pit. Figure 5(d–j) show the SEM and panchromatic CL images of the green MQWs grown on 0, 2, 4, 6, 8, and 10 periods of SL and on low-temperature GaN. Both the SEM image and the panchromatic CL intensity image were recorded at the same location of the green MQWs. The V-pits correspond to dark spots surrounded by a dark halo in the SEM-CL image; this observation indicates the occurrence of non-radiative recombination of the carriers at TDs, which are located at the centres of the V-pits. In addition, the lateral size of the dark halo around the V-pit is much larger than its physical size, suggesting that energetic carriers can be excited over the V-pit potential barrier around the TDs and recombine nonradiatively at TDs.

**Figure 6:**
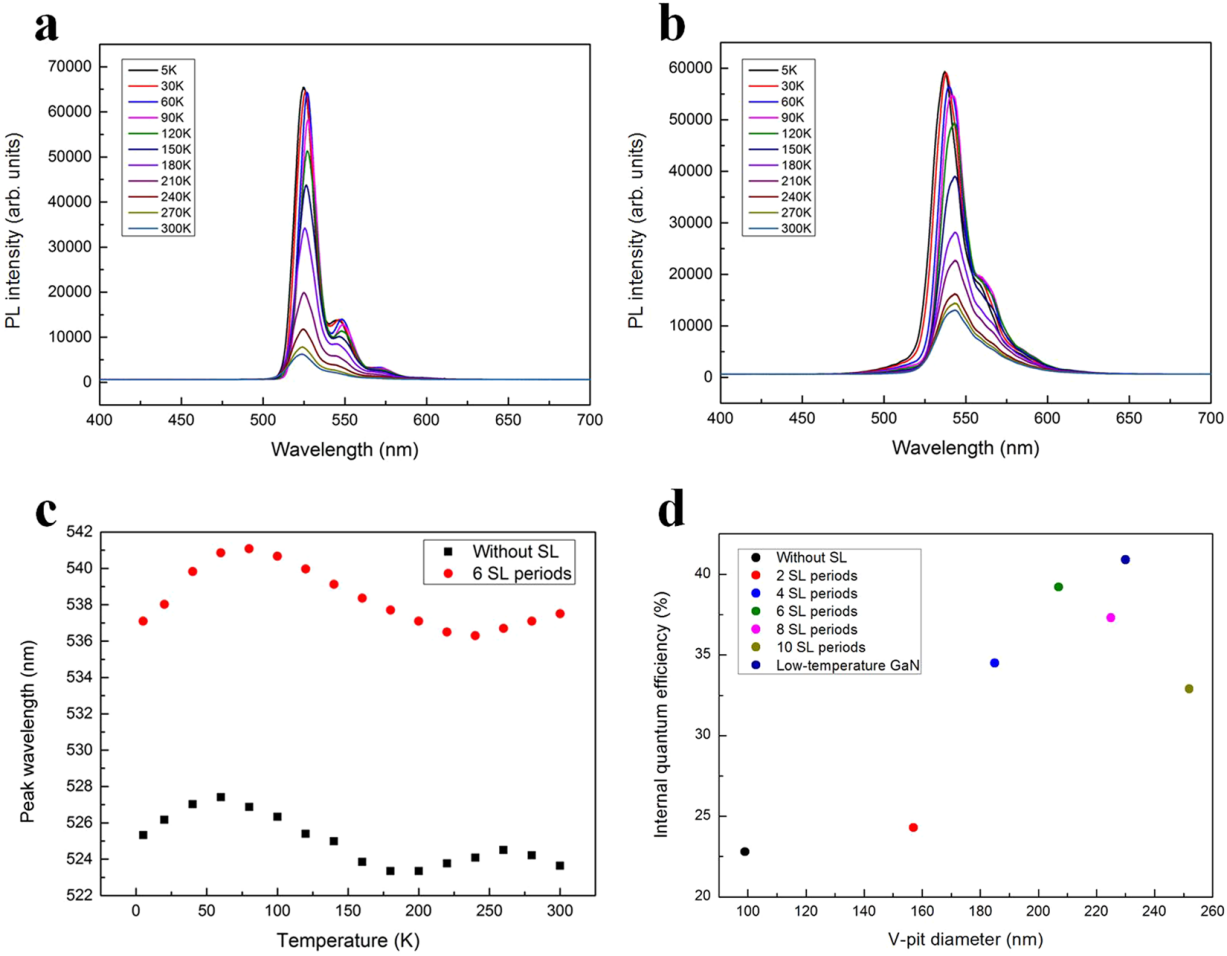
(**a**) Temperature-dependent PL spectra for green MQWs without an SL. (**b**) Temperature-dependent PL spectra for green MQWs grown on 6 SL periods. (**c**) Temperature-dependent peak wavelength of green LEDs without an SL and with 6 SL periods. (**d**) IQE of green MQWs grown on different numbers of SL periods as a function of the V-pit diameter. Variation of the IQE gives rise to the downward parabola. Among these green MQWs grown on various numbers of SL periods, the IQE of the green MQW grown on 6 SL periods is the highest. By replacing 6 periods of SL with low-temperature GaN, the IQE of the green LED is further improved.

After demonstrating that the V-pit potential barrier height and the V-pit diameter are intimately linked and that the effect of number of SL periods on compressive stress of green LEDs, we now turn to analyse the V-pit diameter dependence of the IQE. We measured the PL spectra of the green LEDs grown on 0, 2, 4, 6, 8, and 10 periods of SL and on low-temperature GaN as a function of temperature in the temperature range from 5 to 300 K using a 405 nm semiconductor laser with the power density of approximately 1 kW/cm^2^. To save space, we present only the PL spectra of the green LEDs grown on 0 and 6 periods of SL [Fig. 6(a,b)]. The peak wavelength of the temperature-dependent PL for the two green LEDs samples exhibits ‘S-shaped’ behaviour [Fig. 6(c)], which is a spectral characteristic related to carrier localization in the green LEDs^53–55^.

Assuming that the effect of the NRCs is negligible at the cryogenic temperature of 5 K and that the peak PL quantum efficiency at 5 K is 100%, the ratio of the spectrally integrated PL intensity at 300 K to that at 5 K can be used to estimate the IQE values of green LEDs^56^. Figure 6(d) shows the IQE of the green LEDs grown on various periods of SL as a function of the V-pit diameter. The IQE values of the green LEDs grown on 0, 2, 4, 6, 8, and 10 periods of SL and on low-temperature GaN were determined to be 22.8, 24.3, 34.5, 39.2, 37.3, 32.9, and 40.9%, respectively. Although the compressive stress in the green LED with SL is larger than that in the green LED without an SL, the IQE of the green LED with an SL is higher than that of the green LED without an SL. This result reveals that the V-pit potential barrier height plays a dominant role in determining the quantum efficiency. The IQE of green LEDs increased as the number of SL periods increased from 2 to 6 but decreased as the number of SL periods increased from 6 to 10. As shown in Fig. 5(c), the V-pit potential barrier height was almost saturated as the number of SL periods increased from 6 to 10. The surface coverage ratio values of the V-pits in green MQWs grown on 0, 2, 4, 6, 8, and 10 periods of SL were calculated as approximately 1.2, 3.1, 3.4, 4.0, 4.9, and 6.1%, respectively. As the number of SL periods was increased from 6 to 10, the excessive area loss of the MQW active region resulted in the poor optical output of green LEDs. Additionally, the compressive stress of green LEDs grown on large periods of SL was larger than that of green LEDs grown on small periods of SL. We conclude that the saturation of the V-pit potential barrier height, the excessive area loss of the MQW active region and the increasing compressive stress in green LEDs could play a role in determining the trend of the IQE changes with increasing SL periods.

Akasaka *et al*. suggested that the NRCs near the MQW were greatly reduced by the incorporation of In atoms into the underlying layer, whereas eliminating the NRCs using a low-temperature GaN underlayer is challenging^29^. They found that the emission efficiency of the LED with an InGaN underlayer was much stronger that of the LED with a low-temperature GaN underlayer and that the emission efficiency of the LED with a low-temperature GaN underlayer was almost the same as that of the LED with a conventional high-temperature GaN underlayer. However, we find experimentally that the IQE of green LEDs can be further improved by replacing 6 periods of an In_0.06_Ga_0.94_N/GaN SL with low-temperature GaN. Here, our findings contradict the previous results. The results of our investigation do not support the hypothesis that the InGaN/GaN SL is a possible underlayer for the reduction of NRCs near the MQW. Based on a comparison of our data to the measured size-dependent potential barrier height in Fig. 5(c) and to the measured compressive stress in Fig. 3, we conclude that the increasing V-pit potential barrier height and decreased compressive stress in green LEDs with low-temperature GaN play a critical role in this improvement.

**Figure 7:**
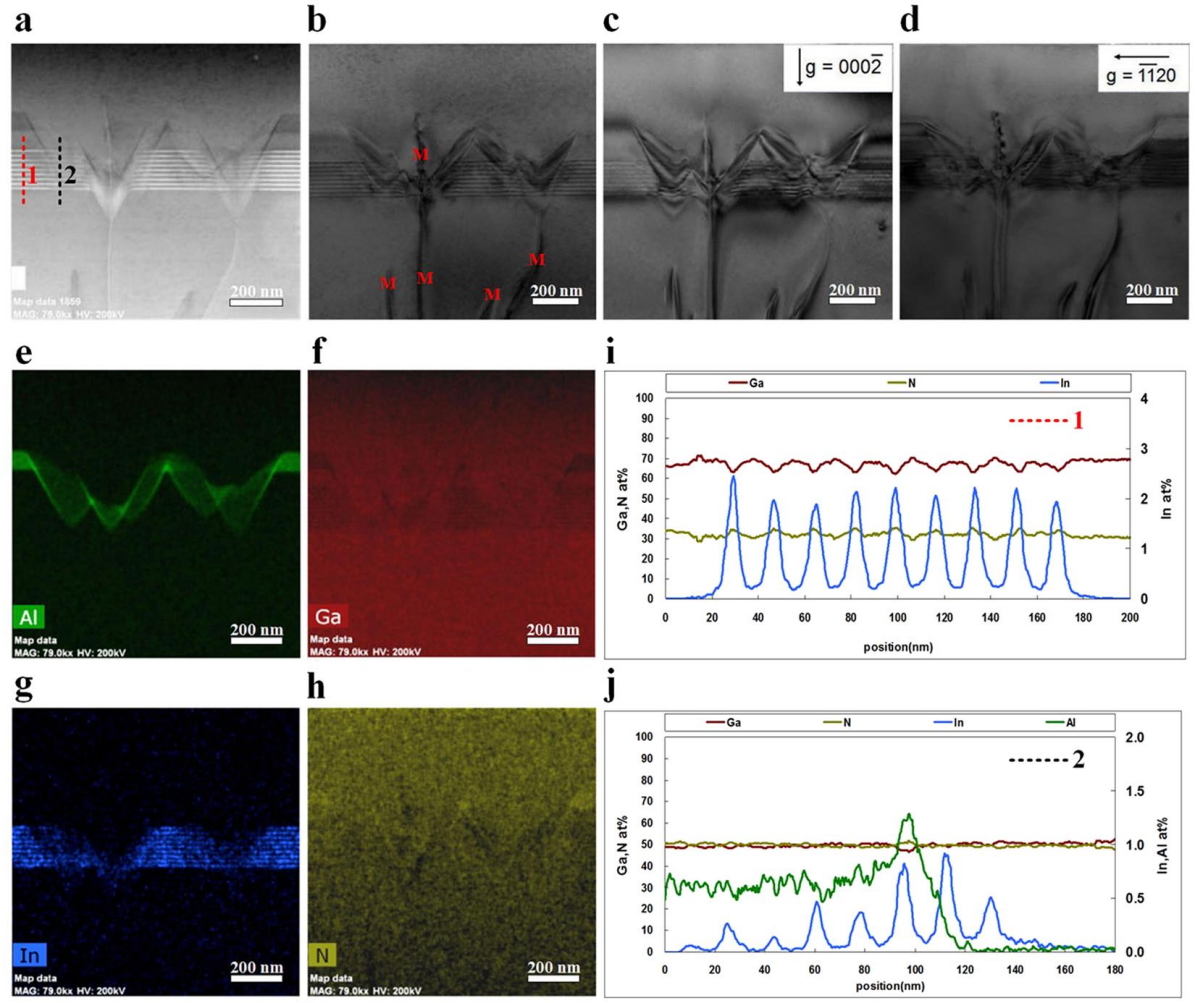
(**a**) Cross-sectional HAADF-STEM image of V-pit-embedded green InGaN/GaN MQW grown on 6 SL periods. The HAADF-STEM image clearly shows the atomic number contrast between In_0.25_Ga_0.75_N well layers (bright contrast) and GaN barrier layers (dark contrast). The V-pits nucleate at TDs. (**b**) Cross-sectional bright-field TEM image showing mixed dislocations running through the apex of the V-pits. Bright-field TEM images of the MQW sample taken at the same area under two-beam diffraction conditions with (**c**) g = (0002) and (**d**) g = (11–20). EDX elemental mapping images showing (**e**) Al, (**f**) Ga, (**g**) In, and (**h**) N maps in green, red, blue, and yellow, respectively. EDX line scans through the MQW sample were obtained in HAADF-STEM mode by performing the scanning vertically along (**i**) surrounding planar MQWs and (**j**) (10–11) sidewall MQWs of the V-pit.

High-angle annular dark field scanning TEM (HAADF-STEM) combined with EDX spectroscopy was performed to further analyse the structural feature of the V-pits. Figure 7 shows cross-sectional bright-field TEM images and EDX analysis of the V-pit-embedded green LEDs grown on 6 SL periods. Multiple V-pits appear in the green MQW, and the bright V-shaped contours represent the exact position of the V-pits in the HAADF-STEM image [Fig. 7(a)]. Figure 7(c,d) show the cross-sectional bright-field TEM images of the sample taken in the same area under two-beam diffraction conditions with g = (0002) and (11–20), respectively. Based on the invisibility criteria g·b = 0, the type of dislocation, namely, screw, edge, or mixed, can be identified. Comparing the two-beam images with g = (11–20) and (0002), we found that each V-pit incorporated a mixed dislocation running through the apex of the V-pit up to the free surface [Fig. 7(b)]. This observation is consistent with the argument advanced in previous reports that V-pits originate at TDs with increased nucleation probability at mixed TDs relative to the pure edge TDs^57^.

EDX elemental (Al, Ga, In, N) mapping images of the V-pit embedded green MQW are presented in Fig. 7(e–h). To reveal the compositional fluctuation along the dotted lines in Fig. 7(a), EDX line-scanning analysis was performed in the HAADF-STEM mode. The presence of Al, Ga, and N signal near the sidewall MQW of the V-pits, as demonstrated by HAADF-STEM and EDX mapping, suggested that these V-pits were subsequently filled in with the p-AlGaN/GaN SL and p-GaN capping layer. These findings unambiguously demonstrate that the (10–11) sidewall MQW of the V-pit exhibited thinner layers and lower In content than the *c*-plane (0001) MQW.

**Figure 8:**
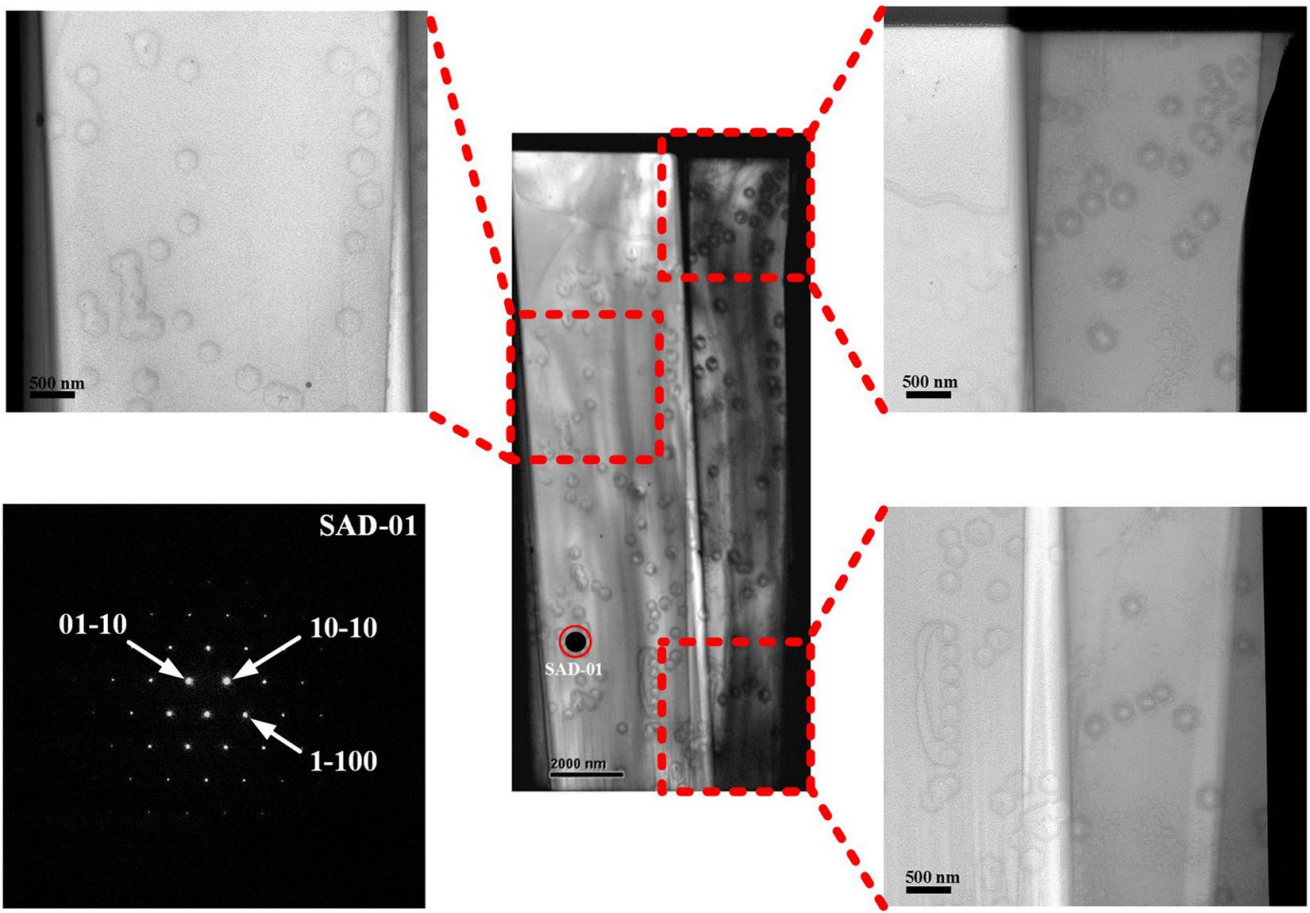
Plan-view TEM images of the green LED specimen including layers of InGaN/GaN MQW, InGaN/GaN SL, and a fraction of n-GaN with SAD pattern of SAD-01. The TD is clearly observed at the centre of hexagonal V-pit.

Figure 8 shows plan-view TEM images of a green LED specimen composed of layers of In_0.25_Ga_0.75_N/GaN MQW, 6 periods of In_0.06_Ga_0.94_N/GaN SL, and a fraction of n-GaN along the *c*-axis growth direction. The pyramidal holes in the plan-view TEM sample surface are V-pits, and each V-pit decorates a TD at the centre of hexagonal base plane. By combining the plan-view TEM image and selected area diffraction (SAD) pattern, we can confirm that the sidewalls of the V-pit are {10–11} planes and the hexagonal side of the V-pit correlates with the intersections of the {0001} and {10–11} planes. The V-pit density in the green LED specimen was approximately 1.25 × 10^8^ cm^−2^ as determined from the plan-view TEM image, consistent with the result obtained by AFM measurements.

**Figure 9:**
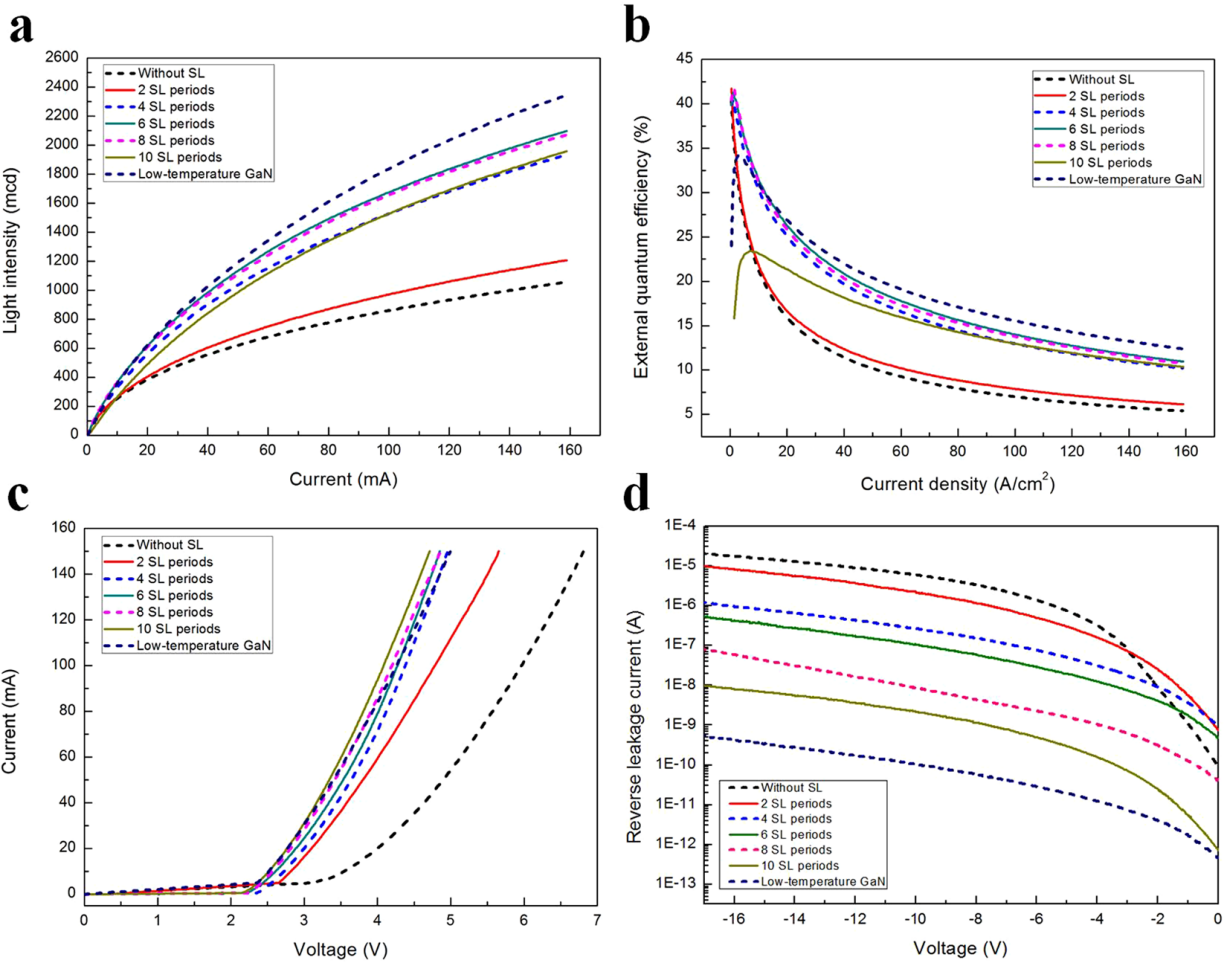
(**a**) Light emission intensity versus current characteristics of green LEDs. (**b**) EQE of green LEDs as a function of injection current. (**c**) Current versus voltage curves of green LEDs. The forward voltage of green LEDs decreases with increasing V-pit diameter. (**d**) Reverse leakage current of green LEDs measured at room temperature.

Equipped with the knowledge of the structural features of the V-pit, we describe how the V-pit diameter affects the electronic and optical properties and efficiency droop of green LEDs. Figure 9 shows the light emission intensity-current-voltage characteristics of green LEDs grown on 0, 2, 4, 6, 8, and 10 periods of SL and on low-temperature GaN. At 20 mA, the light intensities of the green LEDs grown on 0, 2, 4, 6, 8, and 10 periods of SL and on low-temperature GaN were 382, 402, 558, 614, 606, 488, and 618 mcd, respectively [Fig. 9(a)]; the corresponding EQEs of these green LEDs were determined to be 15.9, 16.7, 24.0, 26.3, 25.9, 21.3, and 26.9%, respectively [Fig. 9(b)]. The trend of the EQE changes in these green LEDs is consistent with that of the IQE values measured for these green LEDs. Otsuji *et al*. hypothesized that the electrons injected from the n-type InGaN underlayer are more efficiently captured into the active regions under the forward bias condition, thus enhancing the EL efficiency of LEDs because the band gap energy of the InGaN underlayer is lower than that of the GaN barrier layer^26^. Although the band gap energy of the low-temperature GaN is the same as that of the GaN barrier layer, we note that the EL efficiency of green LEDs is also significantly improved when a low-temperature GaN layer is introduced. Furthermore, the EL intensity of green LEDs can be further improved by replacing InGaN/GaN SL with low-temperature GaN. Accordingly, the improved EL efficiency of green LED with a low-temperature GaN underlayer is not related to the enhancement of the electron capture efficiency.

We also observe changes in the efficiency droop when the V-pit diameter is changed, most clearly observed in Fig. 9(b), where we plot EQE versus the injection current. Generally, the EQE of the GaN-based LED peaks at low-injection current and decreases at the high-injection currents required to operate LEDs; this effect is the so-called efficiency droop. Here, we define the efficiency droop as *η* = (1 − EQE_20*mA*_/EQE_*max*_), where *η* is the efficiency droop ratio, EQE_*max*_ is the maximum efficiency and EQE_20*mA*_ is the efficiency at 20 mA. The resultant efficiency droop values for the green LEDs grown on 0, 2, 4, 6, 8, and 10 periods of SL and on low-temperature GaN were calculated to be 61.5, 59.9, 38.2, 36.2, 34.6, 8.9, and 21.4%, respectively. Figure 9(c) shows a plot of the injection current versus the bias voltage. At 20 mA, the forward voltage of the green LEDs grown on 0, 2, 4, 6, 8, and 10 periods of SL and on low-temperature GaN was 4.03, 3.12, 3.02, 2.91, 2.83, 2.76, and 2.79 V, respectively. Using numerical simulations, we have recently found that V-pits not only enhance the injection of holes into the QWs but also improve the uniformity of the hole density distribution in various QWs^58^. Thus, we expect the forward voltage to decrease with increasing V-pit diameter. Recently, Ren *et al*. reported that the presence of inhomogeneous EL of InGaN/GaN MQW can be ascribed to the increased hole injection in the local area surrounding the V-pit^59^.

Figure 9(d) shows the reverse leakage current of the green LEDs grown on 0, 2, 4, 6, 8, and 10 periods of SL and on low-temperature GaN. At the negative bias of −10 V, the reverse leakage currents of the green LEDs grown on 0, 2, 4, 6, 8, and 10 periods of SL and on low-temperature GaN were 5.99 × 10^−6^, 2.25 × 10^−6^, 2.67 × 10^−7^, 1.06 × 10^−7^, 8.68 × 10^−9^, 2.34 × 10^−9^ and 1.06 × 10^−10^ A, respectively. The reverse leakage current of green LEDs with larger V-pits is significantly reduced. The reverse leakage current of InGaN/GaN LEDs is attributed to the electron tunneling from p-GaN to n-GaN through TDs^60^. The remarkable improvement of the leakage current characteristics of the green LED with larger V-pits can be explained by the effective screening of the TDs. Moreover, the larger V-pits are also more effective for reducing the reverse leakage current of green LEDs due to its higher Poole-Frenkel barrier height^61^.

## Conclusions

We have demonstrated the effects of V-pits on electronic and optical properties and efficiency droop of green LEDs. Our analysis shows that the potential barrier height formed by V-pits around the TDs increased with increasing V-pit diameter, revealing that a larger V-pit could more effectively suppress the non-radiative recombination of carriers at TDs. The emission efficiency of green LEDs first increased and then decreased as the number of SL periods increased from 6 to 12, which can be qualitatively explained by considering the variation of the V-pit diameter-dependent potential barrier and the effective area of the MQW active region. Furthermore, our findings indicate that low-temperature GaN can act as an alternative to the InGaN/GaN SL structure for promoting V-pit formation. The green LED with low-temperature GaN structure can achieve higher emission efficiency than the InGaN/GaN SL structure, suggesting a possible solution for obtaining a further enhancement of the IQE. A decreased forward voltage and an improved efficiency droop were observed for green LEDs with increasing V-pit diameter, owing to the enhanced hole injection. This work demonstrates the potential of the implementation of the optimum V-pits embedded InGaN/GaN SL or low-temperature GaN as a promising underlying layer for high-efficiency green LEDs.
